# Cost-of-illness studies of inherited retinal diseases: a systematic review

**DOI:** 10.1186/s13023-024-03099-9

**Published:** 2024-02-29

**Authors:** Qin Xiang Ng, Clarence Ong, Clyve Yu Leon Yaow, Hwei Wuen Chan, Julian Thumboo, Yi Wang, Gerald Choon Huat Koh

**Affiliations:** 1 Saw Swee Hock School of Public Health, National University of Singapore and National University Health System, Singapore, Singapore; 2 Health Services Research Unit, Singapore General Hospital, Singapore, Singapore; 3 NUS Yong Loo Lin School of Medicine, National University of Singapore, Singapore, Singapore; 4 Department of Ophthalmology, National University Hospital, Singapore, Singapore

**Keywords:** Inherited retinal disease, Retinitis pigmentosa, Blindness, Cost-of-illness, Health economics

## Abstract

**Background:**

While health care and societal costs are routinely modelled for most diseases, there is a paucity of comprehensive data and cost-of-illness (COI) studies for inherited retinal diseases (IRDs). This lack of data can lead to underfunding or misallocation of resources. A comprehensive understanding of the COI of IRDs would assist governmental and healthcare leaders in determining optimal resource allocation, prioritizing funding for research, treatment, and support services for these patients.

**Methods:**

Following PRISMA guidelines, a literature search was conducted using Medline, EMBASE and Cochrane databases, from database inception up to 30 Jun 2023, to identify COI studies related to IRD. Original studies in English, primarily including patients with IRDs, and whose main study objective was the estimation of the costs of IRDs and had sufficiently detailed methodology to assess study quality were eligible for inclusion. To enable comparison across countries and studies, all annual costs were standardized to US dollars, adjusted for inflation to reflect their current value and recalculated on a “per patient” basis wherever possible. The review protocol was registered in PROSPERO (registration number CRD42023452986).

**Results:**

A total of nine studies were included in the final stage of systematic review and they consistently demonstrated a significant disease burden associated with IRDs. In Singapore, the mean total cost per patient was roughly US$6926/year. In Japan, the mean total cost per patient was US$20,833/year. In the UK, the mean total cost per patient with IRD ranged from US$21,658 to US$36,549/year. In contrast, in the US, the mean total per-patient costs for IRDs ranged from about US$33,017 to US$186,051 per year. In Canada, these mean total per-patient costs varied between US$16,470 and US$275,045/year. Non-health costs constituted the overwhelming majority of costs as compared to healthcare costs; 87–98% of the total costs were due to non-health costs, which could be attributed to diminished quality of life, poverty, and increased informal caregiving needs for affected individuals.

**Conclusion:**

IRDs impose a disproportionate societal burden outside health systems. It is vital for continued funding into IRD research, and governments should incorporate societal costs in the evaluation of cost-effectiveness for forthcoming IRD interventions, including genomic testing and targeted therapies.

**Supplementary Information:**

The online version contains supplementary material available at 10.1186/s13023-024-03099-9.

## Introduction

Inherited retinal diseases (IRDs), also called hereditary retinal dystrophies, are a heterogeneous group of orphan genetic disorders that usually lead to severe vision impairment or blindness in infancy, childhood or adulthood [1, 2]. IRDs are caused by mutations in one of more than 317 mapped genes, affecting around 5 to 6 million people worldwide [3] and are a leading cause of blindness among working adults [1].

While health care and societal costs are routinely modelled for most diseases [4, 5], there is a paucity of comprehensive data and cost-of-illness (COI) studies for IRDs. The costs associated with IRDs can have a significant impact on society in various ways. The expenses related to the diagnosis, treatment, and management of IRDs can be substantial [6, 7]. This includes costs associated with outpatient medical visits, genetic testing, specialized imaging, prescription medications, surgeries (such as retinal detachment repair or gene therapy), low vision aids, and rehabilitation services [6, 7]. The need for lifelong monitoring and intervention further adds to the healthcare expenses [6, 7]. In the United States (US) alone, in 2017, the total economic burden of vision loss and blindness amounted to more than US$134 billion for the US population [8]. Moreover, due to the typical onset of IRDs in childhood, its implications extend throughout the individual’s lifespan, affecting both the child and their family [1, 2]. Through focus group discussions with parents of children with vision impairment, several prevailing concerns were identified, including frustrations arising from the absence of a cure for their child’s ocular condition and immense psychosocial challenges experienced by family members who worried about their child being subjected to teasing by peers [9]. These findings collectively underscore that vision impairment not only exerts a profound physical impact, but also threaten the mental health and overall well-being of individuals living with visual impairment and their families.

Specific to the precise estimation of COI of IRDs, such data would help in assessing the economic implications and evaluating the cost-effectiveness and cost–benefit of various interventions, treatments, and healthcare strategies. Individuals with IRDs often require social support and services to cope with their visual impairment and maintain their quality of life. This may include orientation and mobility training, occupational therapy, vision rehabilitation programs, assistive technology and accessibility modifications to living environments [10]. The provision of these services and support systems can thus entail significant costs for society. With the advent of novel gene therapies, current health systems are also not well-configured for large, one-time payments, which is the case of Luxturna (voretigene neparvovec-rzyl) [11]. Luxturna is a novel gene therapy medication used to treat a specific form of inherited retinal disease called Leber congenital amaurosis (LCA) or retinitis pigmentosa (RP) caused by mutations in the *RPE65* gene [11]. In a randomised, controlled clinical trial, 13 of 20 intervention participants (65%) passed multi-luminance mobility testing (MLMT) at the lowest luminance level tested, which measures functional vision at specified light levels, while no control participants did [12]. These improvements in functional vision and visual function were sustained in majority of the participants even after 3 and 4 years [13]. Luxturna offers a viable treatment for a condition previously deemed medically untreatable but it comes with a hefty price tag of around US$850,000 per eye, which may vary depending on insurance coverage and available financial assistance programs [14]. As such, an updated and comprehensive understanding of the COI of IRDs would assist governmental and healthcare leaders in determining resource allocation and prioritizing funding for research, prevention, treatment, and support services.

With this background in mind, this review therefore aimed to systematically review all published COI studies of IRDs and summarize the findings in a standardized manner.

## Methods

In accordance with the latest PRISMA guidelines [15], a systematic review was conducted using Medline, EMBASE and Cochrane Library databases, from database inception up to 30 Jun 2023, to identify COI studies related to IRD. After consultation with an information management specialist, combinations of the keywords (inherited retinal disease) and (cost or economic) were used for the search process, and the full search strategy for the three databases is displayed in Additional file 1: Table S1. The study protocol was registered in the International Prospective Register of Systematic Reviews, also known as PROSPERO, registration number CRD42023452986.

Articles were viewed through Rayyan (Qatar Computing Research Institute, Doha, Qatar, https://www.rayyan.ai), and duplicates were identified and removed. The remaining results were then reviewed independently by three researchers (Q.X.N., C.O., and C.Y.L.Y.) for inclusion. With reference to the Consensus on Health Economic Criteria (CHEC) list [16], the following inclusion criteria were adopted: (1) original study, (2) published or translated into the English language, (3) primarily including patients with inherited retinal diseases (e.g. retinitis pigmentosa), (4) primary study objective is the estimation of the costs of inherited retinal diseases, and (5) with sufficiently detailed methodology for the assessment and evaluation of methodical quality. Commentaries, consensus-based guidelines, case reports, case series, review articles, and conference abstracts were excluded.

Full texts were retrieved for articles which met the inclusion criteria. Two content experts were also consulted for additional references, and references of sources were hand-searched to identify additional relevant articles.

Using a standardized data extraction form, data were extracted into an Excel spreadsheet (Microsoft Corp, Redmond, Washington, United States), including information on the country of origin, type of IRD, study period, data sources, sample size, study design (e.g. retrospective or prospective, prevalence or incidence), cost perspective and reported costs (direct and/or indirect and total). To ensure accuracy, each article was double-coded by two authors and cross-checked for accuracy. For cost data, as different time periods and currencies were used across the studies, local cost values were inflated applying the World Bank’s consumer price index (CPI) [17] to make them equivalent to the cost in 2023, and then converted to US dollars using the FX currency converter for comparison [18]. For a more comprehensive comparison between countries and studies, we also recalculated costs on a “per patient” basis wherever possible.

## Results

A total of 777 articles were initially identified as potentially relevant. After removing 35 duplicates/triplicates, 742 studies remained for screening based on title and abstract. Out of these, 695 papers were excluded as they did not pertain to COI studies of IRD. Among the remaining 47 articles, 38 studies did not meet the inclusion criteria defined a priori. Consequently, this review analyzed a total of 9 articles, considering their study characteristics and cost data.

**Fig. 1:**
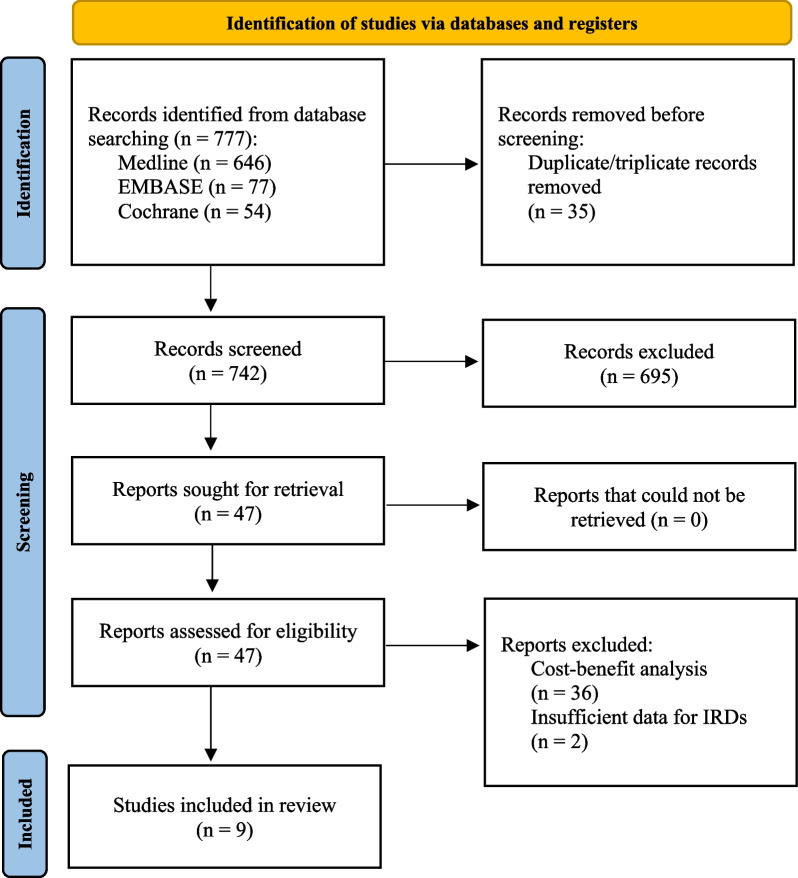
PRISMA flowchart showing the study abstraction process

The study abstraction process is illustrated in Fig. 1.

Four studies were from the United States (US) [7, 19–21] (one with Canada [7]), and one each from Australia [22], Denmark [23], Japan [24], Singapore [25], and the United Kingdom (UK) with Republic of Ireland (RoI) [6]. The study characteristics and salient study findings are detailed in Table 1 and 2 respectively. To enable comparison across countries and studies, all costs were standardized to US dollars, adjusted for inflation to reflect their current value, and recalculated on a “per patient” basis wherever possible.

**Table 1:** Characteristics of COI studies for IRDs, arranged by chronological order (n = 9)

Study (year of costing)	Country	Type of IRD	Data source	Sample size	Study design	Perspective	Mean total costs per year	Mean health costs per year (%)	Mean non-health costs per year (%)
Aziz et al., (2021) [19]	United States (US)	Stargardt disease	Insurance claims	472,428	Retrospective, prevalence-based	Healthcare system	NR	US$105.58/pt	NR
Chay et al., (2023) [25]	Singapore	Group of IRDs (63.2% with retinitis pigmentosa; median age of symptom onset was 32 years)	Multiple data sources	500	Prospective, prevalence-based	Societal	S$48,810,000 (= US$36,027,137) S$9382/pt (= US$6926.36/pt)	S$6,200,000 (= US$4,577,218) S$1194/pt (= US$881.48/pt) (12.1%)	S$42,590,000 (= US$31,442,536) S$8188/pt (= US$6044.88/pt) (87.9%)
Frick et al., (2012) [20]	United States	Retinitis pigmentosa	Insurance claims	2990	Retrospective, prevalence-based	Healthcare system	NR	US$14,988/pt	NR
Galvin et al., (2020) [6]	Republic of Ireland (RoI)	Group of 10 IRDs (achromatopsia, best disease, choroideremia, cone dystrophy, cone-rod dystrophy, Leber congenital amaurosis, retinitis pigmentosa, Stargardt disease, Usher syndrome and X-linked retinoschisis)	Multiple data sources	1521	Retrospective, prevalence-based	Societal	£42,600,000 (= US$53,937,990) US$24,107-US$40,718/pt	£1,900,000 (= US$2,405,685) (4.5%)	£40,700,000 (= US$51,532,305) (95.5%)
	United Kingdom (UK)			20,815			£523,300,000 (= US$6662,576,295) US$21,658-US$36,549/pt	£25,000,000 (= US$31,653,750) (4.8%)	£498,300,000 (= US$630,922,545) (95.2%)
Gong et al., (2021) [7]	United States	Group of 14 IRDs (achromatopsia, Bardet-Biedl Syndrome, best disease, blue cone monochromacy, choroideremia, cone dystrophy, cone-rod dystrophy, Leber congenital amaurosis, Leber’s hereditary optic neuropathy, retinitis pigmentosa, rod-cone dystrophy, Stargardt disease, Usher syndrome and X-linked retinoschisis)	Multiple data sources	51,325–122,007	Retrospective, prevalence-based	Societal	US$13,414mil-US$31,797.4mil US$33,017-US$186,051/pt	US$963.8mil- US$2,216.8mil (7%)	US$12,450.2mil- US$29,580.6mil (93%)
	Canada (CA)			5,841–23,891			CAN$1,637.8mil- CAN$6,687.5mil (= US$1,236.0mil- US$5,046.9mil) US$16,470-US$275,045/pt	CAN$37.8mil- CAN$144.3mil (= US$28.5mil- US$108.9mil) (2%)	CAN$1,600mil- CAN$6,543.2mil (= US$1,207.5mil- US$4,938.0mil) (98%)
Kessel et al., (2022) [23]	Denmark	Group of IRDs (childhood-onset; not specified)	Multiple data sources	412	Retrospective, prevalence-based	Healthcare system	NR	(Overall) €1,488/pt (= US$1,619.6/pt) (Age 0–10) €1,145/pt (= US$1,246.3/pt) (Age 11–20) €1,409/pt (= US$1,533.6/pt) (Age 21–30) €1,520/pt (= US$1,654.4/pt) Age (30–48) €1,867/pt (= US$2032.1/pt)	NR
Schofield et al., (2023) [22]	Australia	Group of IRDs (not specified)	Patient and caregiver survey	94 (patient) 30 (carer)	Prospective, Markov modelling, prevalence-based	Societal	AUS$5.2mil/pt (= US$3,452,140/pt) (Lifetime) AUS$781mil- AUS$1,560mil (= US$518.5mil- US$1,035.6mil) (For whole society)	AUS$690,725/pt (= US$458,554/pt) (Lifetime, 13%)	AUS$4,509,275/pt (= US$2,993,586/pt) (Lifetime, 87%)
Dong et al., (2021) [21]	United States	Choroideremia	Insurance claims	199	Retrospective, prevalence-based	Healthcare system	NR	US$15,372/pt	NR
Watanabe et al., (2023) [24]	Japan	Retinitis pigmentosa (mean age of onset 11.2 years)	Patient survey	122	Prospective, cross-sectional, prevalence-based	Societal	US$20,833/pt US$1,766,013/pt (Lifetime)	US$2176/pt US$184,501/pt (Lifetime, 10%)	US$18,657/pt US$1,581,554/pt (Lifetime, 90%)

Ranges reflect minimum–maximum values. Abbreviations: CA, Canada; IRD, inherited retinal disease; NR, not reported; pt, patient; RoI, Republic of Ireland; UK, United Kingdom; US, United States

**Table 2:** Components of health-related and non-health-related costs for IRDs

Study	Health-related costs	Non-health-related costs
Aziz et al. [19]	Medical examination and evaluation (i.e. cost of doctor and other diagnostics)	NR
Chay et al. [25]	Medical costs, assistive technologies, carer cost	Expected productivity loss
Frick et al. [20]	Inpatient hospital admissions, inpatient hospital days, emergency department visits, outpatient physician visits, prescription drug fills	NR
Galvin et al. [6]	Primary/secondary care, diagnostic tests, pharmaceuticals, vitamins and supplements, medical research	Wellbeing costs, productivity loss, deadweight losses, informal care costs
Gong et al. [7]	Primary/secondary care, diagnostic tests, pharmaceuticals, vitamins and supplements	Wellbeing costs, productivity loss, deadweight losses, informal care costs
Kessel et al. [23]	Primary care, outpatient services, inpatient admissions, prescription medication	NR
Schofield et al. [22]	Direct healthcare costs such as use of health products such as pharmaceuticals (unspecified)	Productivity loss, government service and NDIS costs, informal care costs
Dong et al. [21]	Inpatient admissions, outpatient visits, ED visits, pharmacy prescriptions	NR
Watanabe et al. [24]	Healthcare services, paid care, medications and supplements, vision aids	Productivity loss, financial aid received

Abbreviations: NDIS, national disability insurance scheme; NR, not reported

All the studies used a prevalence-based COI approach, which estimates the total costs of a disease within a specified time period, typically 1 year [6, 7, 19–25]. In two studies [6, 7], wellbeing costs were estimated using the World Health Organization (WHO) burden of disease methodology, which quantified the impact of pain, suffering, and premature mortality by measuring disability-adjusted life years (DALYs) [26].

As seen in Tables 1 and 2, there is a wide range of costs in the literature. In Singapore, the mean total cost per patient was roughly US$6,926/year. In Japan, the mean total cost per patient was US$20,833/year. In the UK, the mean total cost per patient with IRD ranged from US$21,658 to US$36,549/year. In contrast, in the US, the mean total per-patient costs for IRDs ranged from about US$33,017 to US$186,051 per year. In Canada, these mean total per-patient costs varied between US$16,470 and US$275,045/year. In terms of relative contribution (Table 1), non-health costs constituted the overwhelming majority of costs (87 to 98%) as compared to health costs [6, 7, 22, 24, 25]. Health expenses encompass a range of components, such as health-related costs including utilizing primary and secondary care services, diagnostic examinations, pharmaceutical drugs, nutritional supplements, and investments in medical research [6, 7, 19, 20, 22–25], while the non-health-related costs mainly pertain to wellbeing costs (DALYs), productivity loss (referring to loss in productivity due to illness or disability), and deadweight losses (referring to loss of economic efficiency that can occur when equilibrium for a good or service is not achieved or is not achievable).

## Discussion

In this review, a total of nine contemporary studies [6, 7, 19–25] were examined and they highlighted a substantial economic burden associated with inherited retinal diseases (IRDs). These studies demonstrate not only the direct healthcare costs but also the expansive societal costs arising from IRDs.

In the context of healthcare costs, in the longitudinal study by Kessel et al. [23], among individuals with childhood onset IRDs, healthcare costs (consisting of hospital, primary care, prescription medications and home care costs) were found to be approximately 40% higher compared to a sample from the general Danish population that was matched for age and sex. In terms of other costs, the microsimulation modelling study done in Australia by Schofield et al. [22] found that the lifetime cost of IRD was more than US$3.4 million per person, and 87% of the total costs were societal. This is perhaps unsurprising as vision loss itself has several known consequences for the affected individual, including an elevated risk of poverty, diminished quality of life, limited employment prospects and increased financial burdens associated with informal caregiving [27, 28], although the unexpected finding of lower costs associated with injuries in the visually impaired cohort in the study by Kessel et al. [23] is more likely the result of a lower likelihood to seek medical attention rather than a reduced susceptibility to injuries.

Therefore, it follows that it is important to consider non-health costs. When evaluating the cost-effectiveness of interventions for individuals with IRDs, both health costs and non-health costs should be taken into account. As reflected by the percentages in Table 1, non-health costs contribute a significant proportion compared to health costs. Non-health costs encompass various factors that extend beyond direct financial expenses. While some studies focused solely on productivity costs [24, 25], others also considered wellbeing costs [6, 7]. It would not be fair to compare all studies that incorporate non-health costs, as the specific types of costs assessed vary. For instance, a study in Singapore by Chay et al. [25] examined productivity costs exclusively, whereas other papers [6, 7] took into account both productivity costs and wellbeing costs. In the area of health economics, although most researchers acknowledge the importance of incorporating productivity loss as a component of societal cost [4, 5, 29], opinions may differ when it comes to including wellbeing costs in these studies. The question arises as to whether it should be standard practice to consistently incorporate wellbeing costs in COI evaluations of IRDs. While productivity loss can be more straightforward to quantify, wellbeing costs capture the broader impact on individuals’ quality of life and overall well-being. However, standardizing the inclusion of wellbeing costs in COI studies presents certain challenges. It requires defining and measuring wellbeing costs reliably across studies, which can be complex due to the subjective nature of assessing well-being. In light of these considerations, the decision to include wellbeing costs in COI studies evaluating IRDs should be weighed carefully, balancing comprehensiveness with practicality, to ensure that the chosen methodology effectively captures the pertinent societal costs associated with IRDs.

With regard to the study methodology, all the studies used data specifically from IRD patients (and not other vision disorders), and the majority of available studies relied on primary and linked administrative data [6, 7, 19–21, 23, 25] to estimate the COI related to IRDs, while others used patient and caregiver surveys [22, 24]. Insurance claims might provide more precise healthcare utilization data but may not capture all societal costs, while survey data can offer insights into the patient and caregiver experience, including aspects like out-of-pocket expenses and quality of life impacts. Several studies [19–22] considered health costs only (i.e. from a healthcare system perspective) as opposed to studies that also considered the societal perspective [6, 7, 22, 24, 25]. Given that individuals with IRDs tend not to heavily utilize healthcare services [6], assessing the cost of illness from a non-health perspective, which includes the economic impact on other non-health (e.g. social) sectors, would be a more comprehensive and representative approach.

The variation in estimated costs across the studies reviewed may stem from several factors. First, differences in healthcare systems across countries may contribute to the cost differences. For instance, the UK predominantly operates a public healthcare system, while the US relies more heavily on a larger private sector system [30]. Consequently, healthcare expenditure and the allocation of costs differ significantly between countries. Second, the organization of healthcare services in the countries also differ; US healthcare system primarily consists of private companies and the Canadian healthcare system primarily operates as a publicly funded system [31]. This would affect the accessibility of healthcare services, with profound health inequities experienced by certain vulnerable groups [30, 31]. In the UK, the cost per patient with IRD ranged from US$21,658 to US$36,549/year [6]. In contrast, in the US, the per-patient costs for IRDs ranged from about US$33,017 to US$186,051 per year [7]. In Canada, these per-patient costs varied between US$16,470 and US$275,045/year [7]. Third, differences may also arise due to differences in the age of onset and severity of disease, which was not reported in the majority of studies reviewed. Stargardt disease, for example, can start in childhood or adulthood; an older age of symptom onset is thought to be associated with better vision while a longer duration of symptoms is associated with worse vision [32]. Hence, the age of onset and progression of vision loss would affect COI calculations. Schofield et al. [22] compared the costs for IRD patients who were legally blind and those with better vision, and they found that although healthcare costs were slightly higher for those with better vision, overall costs were substantially higher (more than twice) for those who were legally blind. Fourth, as mentioned earlier, IRDs are a heterogeneous group of diseases, and the costs associated with each can vary significantly based on a range of factors including the severity of the condition, the availability and cost of treatment options, and the level of support required by patients. Unfortunately, all the studies that studied IRDs only looked at the collective impact and burden of IRDs, without detailed breakdown of these costs on a per-patient basis for each IRD, making it hard to make specific inferences [6, 7, 20, 21, 23]. Certain IRDs stand out and may have a greater COI, such as Usher Syndrome, which not only affects vision but also causes deafness [33], and Stargardt Disease, which may have an early onset and progressive vision loss [32]. Last but not least, some of the studies reviewed were constrained by data limitations, particularly in their estimates of income losses, which were often based on comparisons with the average income of the general population [6, 7, 23]. A more precise method would involve directly comparing individuals with diagnosed IRDs and their caregivers to a matched group from the general population, which shares similar characteristics [22, 23, 25]. This approach would better estimate the potential income and tax contributions these individuals might have made had they not been affected by IRDs.

It is recognizable that the impact of IRDs on employment and career progression is a factor contributing to the progressive loss of income over the lifespan of affected individuals [22]. In the study by Galvin et al. [6], the likelihood of individuals with IRDs being employed was 55.7% and 40.2% lower, in the RoI and the UK respectively, compared to the general population. Schofield et al. [22] suggests that enhancing employment support for people with IRDs could significantly improve societal outcomes. Another example is observed in Singapore, where the employment rates of individuals with IRDs were on par with the general population [25], although there might be certain selection biases as the IRD cohort was fairly young (mean age 47.90 years) and 51.6% had unimpaired central visual acuity. However, despite comparable employment rates—67.4% in the IRD group versus 70.7% in the age- and gender-matched general population—individuals with IRDs typically earn 26% less [25]. The disparities in social support elsewhere and differences in income earnings highlight a potential area for intervention. In economies with more robust support systems, individuals with IRDs may be able to secure employment, and societal structures and policies can positively influence employment outcomes for people with visual disabilities [22, 25].

### Study strengths and limitations

To the best of our knowledge, this is the first systematic review on the COI of IRDs. The review highlights the multifaceted nature of these costs. IRDs not only impose significant healthcare costs but also extend to broader societal costs, including an elevated risk of poverty, reduced productivity and a greater need for social support services. Moreover, the lifetime burden of these diseases is substantial, given their potential onset in the first and second decade of life and the consequent lifelong implications for the individual and their families. Therefore, in evaluating the cost-effectiveness of various interventions and treatments for IRDs, it is necessary to consider both the health-related costs and the non-health costs.

While most studies reported annual patient costs, few reported annual lifetime costs per patient [22, 24]. Although the former offers a macroeconomic perspective of whole societal impact, cost-effectiveness analyses (CEAs) require a more focused, patient-centric view of costs. Using incremental cost-effectiveness ratio analysis, a recent study suggested that Luxturna is in fact cost-effective when compared with standard care [14]. The authors used a lifetime horizon, excluded indirect costs, and set a threshold of US$150,000 per quality-adjusted life-year [14]. For CEAs in particular, the annual or lifetime costs per patient are what is needed.

Limitations of the present review include the general lack of estimates of population prevalence for IRDs, average life expectancy of patients with IRDs, discrepancies in diagnosis and definitions especially since IRDs are a heterogeneous and complex group of conditions [2, 3]. COI findings must also be interpreted in the context of a particular country’s prevailing healthcare policies and funding structure as previously discussed. Regrettably, the studies reviewed also could not exclude the possibility of ascertainment bias in their prevalence estimates.

## Conclusion

In conclusion, the review highlights that IRDs impose a disproportionate societal burden outside health systems, predominantly attributed to lower employment rates among both patients and caregivers, as well as increased reliance on social support. It is vital for governments and the relevant authorities to incorporate non-health (societal) costs in the evaluation of cost-effectiveness for forthcoming IRD interventions, including genomic testing and targeted therapies, and future studies should also calculate annual (or lifetime) costs per patient to facilitate such cost-effectiveness analyses. Continued research funding and the implementation of nuanced, tailored policies are also critical for mitigating the socioeconomic impact of IRDs. This may necessitate the allocation of research funds towards identifying remaining unidentified causal genes, ongoing research for treatment and therapy development, improved accessibility to genetic testing and counselling, policy reassessment and development concerning reimbursement methods for IRDs in terms of care and treatment, and clear management pathways for individuals with IRDs.

### Supplementary Information

**Additional file 1. Table S1.** Full search strategy for the various databases.

## Data Availability

The authors confirm that the data supporting the findings of this study are available within the article and its supplementary material.
