# Supplementary material for: Cost-of-illness studies of inherited retinal diseases: a systematic review
Source: Orphanet J Rare Dis. 2024 Feb 29;19:93. doi: 10.1186/s13023-024-03099-9 (PMC10905859; doi:10.1186/s13023-024-03099-9)
Supplement: Supplementary file 1 — Additional file 1. Table S1. Full search strategy for the various databases. [file 13023_2024_3099_MOESM1_ESM.docx]

**Supplementary Material**

Table S1. Full search strategy for the various databases

**MEDLINE**

| Inherited Retina Disease | exp Retinal Diseases/ or exp Retinitis Pigmentosa/ or exp Eye Diseases, Hereditary/ or ('inherited retinal disease*' or 'IRD' or 'IRDS' or 'inherited retinal disorder' or 'hereditary retinal disease*' or 'hereditary eye disease*').ti,ab |
| --- | --- |
| Cost of Illness | (cost? adj2 (illness or disease or sickness)).tw.  or  (burden? adj2 (illness or disease? or condition? or economic*)).tw.  or  ("quality‐adjusted life years" or "quality adjusted life years" or QALY?).tw.  or  exp Quality-Adjusted Life Years/  or  exp "Cost of Illness"/  or  exp Health Expenditures/  or  ("out‐of‐pocket" adj2 (payment? or expenditure? or cost? or spending or expense?)).tw.  or  ((adjusted or "quality‐adjusted") adj2 year?).tw.  or (expenditure? adj3 (health or direct or indirect)).ti,ab. |

**EMBASE**

| Inherited Retinal Disease | 'retinitis pigmentosa'/exp OR 'inherited retinal disease*':ti,ab OR 'ird':ti,ab OR 'irds':ti,ab OR 'inherited retinal disorder':ti,ab OR 'hereditary retinal disease*':ti,ab OR 'hereditary eye disease*':ti,ab |
| --- | --- |
| Cost of Illness | (cost* NEXT/2 (illness OR disease OR sickness)):ti,ab or (burden? NEXT/2 (illness OR disease? OR condition? OR economic*)):ti,ab or 'quality‐adjusted life years':ti,ab OR 'quality adjusted life years':ti,ab OR qaly?:ti,ab or 'quality adjusted life year'/exp or 'cost of illness'/exp or 'health care cost'/exp or ('out‐of‐pocket' NEXT/2 (payment? OR expenditure? OR cost? OR spending OR expense?)):ti,ab or ((adjusted OR 'quality‐adjusted') NEXT/2 year?):ti,ab or (expenditure? NEXT/3 (health or direct or indirect)).ti,ab. |

**Cochrane**

**
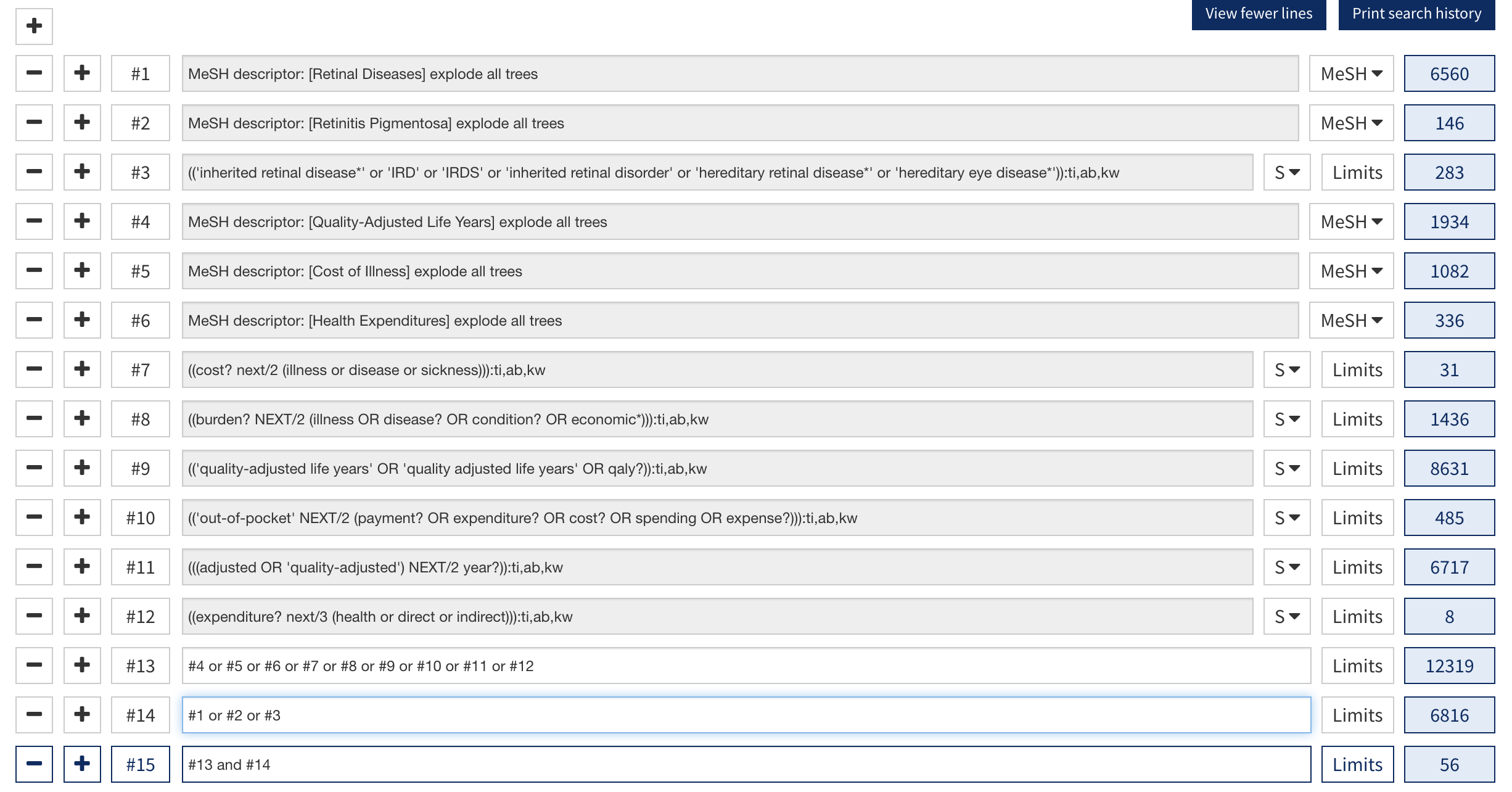
**

Abbreviations:

ti, ab = title, abstract

exp = exploded

adj = adjacent

tw = text word

NEXT/2 = search terms specified are within 2 words of each other and in the order entered

kw = keyword
